# Evans Syndrome Associated with Pregnancy and COVID-19 Infection

**DOI:** 10.1155/2020/8862545

**Published:** 2020-08-18

**Authors:** Gayathri Vadlamudi, Leah Hong, Madhurima Keerthy

**Affiliations:** Department of Obstetrics and Gynecology, Henry Ford Hospital, Detroit, MI, USA

## Abstract

**Background:**

Evans syndrome (ES) is a chronic autoimmune disease characterized by autoimmune hemolytic anemia along with immune thrombocytopenic purpura. Few case reports of ES in pregnancy have been published, and ES may be difficult to distinguish from other diagnoses more common in pregnancy. Guidelines for treatment of ES are not well-defined.

**Case:**

A 23-year-old multigravid woman in active labor was found to have severe anemia and thrombocytopenia. She was diagnosed with ES and started on immunosuppressive treatments for persistent immune thrombocytopenic purpura. In the postpartum period, she was found to have coronavirus (COVID-19) infection and acute pulmonary embolism.

**Conclusion:**

Evans syndrome is a challenge to diagnose in pregnancy and poses important considerations for intrapartum and postpartum management.

## 1. Introduction

Evans syndrome (ES) is a chronic autoimmune disease characterized by autoimmune hemolytic anemia (AIHA) with immune thrombocytopenic purpura (ITP) [1]. Clinical features include pallor, weakness, fatigue, jaundice, petechiae, ecchymosis, and epistaxis. Diagnosis is made by a positive direct antiglobulin test (DAT) in the setting of hemolytic anemia.

The incidence of ITP alone is 1-5 cases per 10,000 pregnancies [2]. Although the incidence of ES in pregnancy is not well known, it has been diagnosed in 1.8%-10% of patients with ITP. The majority of patients with ES have onset in childhood, and 60%-70% of patients with ES are women [1].

In pregnant patients with anemia and thrombocytopenia, it is important to differentiate ES from causes that are more common in pregnancy. We present a case report of a patient with ES diagnosed during pregnancy, her clinical course, treatments, and complications during the postpartum period.

## 2. Case Presentation

A 23-year-old gravida 2, para 1, with no known medical history presented at 38 weeks of gestation with spontaneous rupture of membranes, contractions, and blood-tinged discharge. She reported uncomplicated prenatal care; however, no records could be obtained.

On admission, the patient's vital signs were within normal limits. She was 8 cm dilated on the cervical exam. Due to her desire for pain control, and in anticipation of expeditious delivery in a multiparous patient, anesthesia was consulted for neuraxial analgesia. A review of her obstetric history revealed a prior uncomplicated vaginal delivery at 38 weeks of gestation. Laboratory studies from that admission revealed hemoglobin of 12.5 g/dL and platelet count of 223 K/*μ*L. She had no known history of thrombocytopenia, so an epidural was placed prior to obtaining complete blood count results, which was in accordance with hospital policy.

Laboratory testing (Table 1) resulted afterwards and was notable for hemoglobin of 7.1 g/dL, hematocrit of 22.1%, mean corpuscular volume of 74.7 fL, and platelets of <10 K/*μ*L. Upon further questioning, the patient reported a history of ecchymosis and an episode of epistaxis 2 weeks prior. She reported no history of heavy menstrual bleeding, hemarthrosis, or bleeding gums. On examination, no pallor, ecchymosis, or organomegaly was detected. There was a concern for hemodilution of the blood sample due to the location of the blood draw or laboratory error, so the blood was redrawn.

While awaiting repeat laboratory results, the patient delivered a male neonate by spontaneous vaginal delivery. Neonatal laboratory studies were normal with a hemoglobin of 18.8 g/dL and platelet count of 245 K/*μ*L. Total quantitative blood loss during delivery was 400 mL. Epidural was removed immediately post delivery. Subsequently, repeat laboratory tests confirmed the initial results, with hemoglobin of 5.6 g/dL, hematocrit of 17.7%, and platelets of <10 K/*μ*L. Further laboratory studies (Table 1) showed no marked abnormalities in coagulation profile or hepatic panel results. Iron and B12 deficiencies were noted.

**Figure 1:**
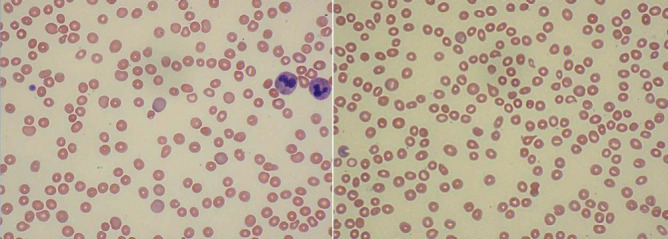
Peripheral blood smear showing microcytic, slightly hypochromic anemia with anisocytosis and polychromasia, thrombocytopenia, and slight leukocytosis. Teardrop cells, reticulocytes, and small spherocytes are present. No overt schistocytes, fragmentation of red blood cells, overt dysplasia, or presence of blasts noted.

Peripheral blood smear (Figure 1) revealed microcytic anemia with anisocytosis, polychromasia, and thrombocytopenia. Teardrop cells, reticulocytes, and small spherocytes were present. No overt schistocytes or fragmentation of red blood cells was noted. There was no concern for thrombotic thrombocytopenic purpura.

Antibody screen was positive for warm autoantibodies. DAT was positive for anti-immunoglobulin G and anti-complement antibodies. The diagnosis of ES was made based on the positive DAT in the setting of hemolytic anemia.

**Figure 2:**
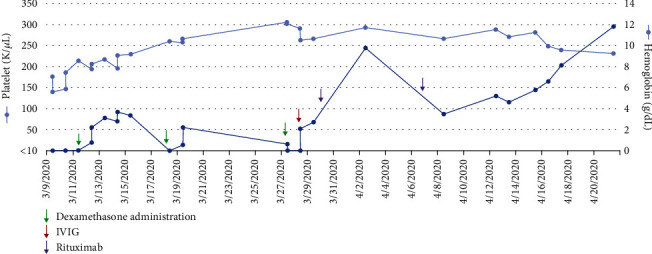
Platelet and hemoglobin values over time. Times of therapy noted by colored arrows. Initial course of steroids with good response, however subsequent decline in platelet count, requiring further treatment with additional steroids, IVIG, and rituximab. Abbreviations: IVIG: intravenous immunoglobulin.

Hematology was consulted, and transfusion of 2 units of red blood cells and 1 pack of platelets was recommended. The patient also received 1 gram of intravenous iron dextran. She was treated with oral dexamethasone, 40 mg daily for 4 days, with a subsequent rise in platelets to 55 K/*μ*L (Figure 2). She was discharged on postpartum day 4 with a plan for weekly outpatient laboratory studies and oral prednisone (60 mg daily). She was also started on folate (1 mg daily) and B12 (1000 mcg monthly) supplementation.

On postpartum day 9, follow-up laboratory studies revealed a platelet count of <10 K/*μ*L. The patient received a second 4-day course of oral dexamethasone, 40 mg daily, with a good response. Her platelet count rose to 55 K/*μ*L. On postpartum day 18, laboratory results showed the platelet count decreased to 16 K/*μ*L, and she received a third course of dexamethasone, 20 mg daily for 4 days, and intravenous immunoglobulin (IVIG), 1 mg/kg for 2 days. Repeat DAT remained positive for warm autoantibodies. Given the poor response to steroids, rituximab therapy was initiated. On postpartum days 21 and 28, the patient received 2 doses of rituximab (375 mg/m^2^ weekly).

**Figure 3:**
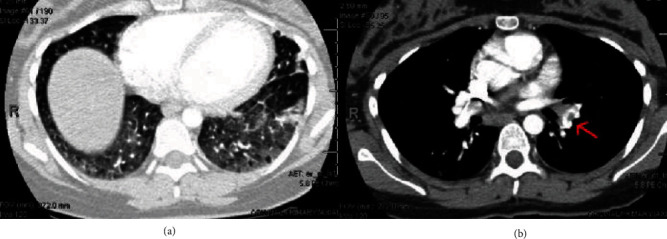
Computed tomography of the chest demonstrating diffuse ground-glass opacities (a) throughout both lungs, which can be seen with COVID-19 pneumonia. Acute pulmonary emboli (b) identified in segmental branches of lingula and left inferior lobar pulmonary artery extending into segmental branches of left lower lobe, with filling defect noted by red arrow.

On postpartum day 34, the patient presented to the emergency department with chest pain and shortness of breath. The evaluation was notable for elevated D-dimer, and a computed tomography scan was obtained due to suspicion of pulmonary embolism. Imaging revealed ground-glass opacities and an acute pulmonary embolism (Figure 3). Notably, lower extremity dopplers during initial admission showed no venous thromboses. The patient was started on anticoagulation with high-intensity heparin infusion. The patient reported that her mother had tested positive for the recently emerged novel coronavirus (COVID-19). Given her symptoms, sick contact status, and imaging findings, the patient was tested for COVID-19. Nasopharyngeal specimen testing for detection of the 2019 novel coronavirus ribonucleic acid by real-time polymerase chain reaction was positive. The platelet count on readmission was 131 K/*μ*L and remained stable over 200 K/*μ*L. Rituximab infusion was held for several weeks due to active COVID-19 infection and stable platelet count.

## 3. Discussion

ES is an autoimmune disorder with autoantibodies against red blood cells and platelets leading to the development of AIHA and ITP. Diagnosis of ES in pregnancy must be differentiated from other conditions more commonly associated with pregnancy. This patient's clinical presentation and laboratory studies were not suggestive of preeclampsia, disseminated intravascular coagulation, or hemolysis, elevated liver enzymes, or low platelet count (HELLP) syndrome. Blood pressure, liver function tests, and coagulation profile on admission were normal. Gestational thrombocytopenia was thought to be unlikely due to the extremely low platelet count.

In a literature search of cases of ES in pregnancy, a 2010 review article identified a total of 14 pregnancies, with data available for 9 cases [2]. Case reports of an additional 5 cases have since been published [2–5]. Of these 14 pregnancies for which data is available, 5 were complicated by preeclampsia, 3 by postpartum hemorrhage, and 1 with placental abruption. Two pregnancies were associated with stillbirth, one of these with a fetal intracranial subdural hematoma and the other with an erythroblastic fetus. One neonate showed evidence of hemolysis 2 months postpartum that spontaneously improved.

Guidelines for the treatment of ES are not well-defined. Steroids are often considered first-line therapy, with the use of IVIG in severe cases. Second-line therapies include immunosuppressive agents such as rituximab, mofetil mycophenolate, and cyclosporine. In the series of cases described above, 5 of 14 patients improved with steroid treatment exclusively and 5 received IVIG. One patient underwent plasma exchange in addition to immunosuppressive therapies and 5 ultimately underwent splenectomy. As with the patient described in this case report, many of these cases demonstrated persistent relapsing ITP, while hemoglobin stabilized early in presentation.

During intrapartum management of a patient with ES, regional anesthesia must be managed carefully. According to the “Practice Guidelines for Obstetric Anesthesia” published by the American Society of Anesthesiologists [6], routine evaluation of platelet count prior to epidural placement is not necessary in healthy patients. This case reinforces the need in urgent situations to balance the patient's desires for pain control with safety concerns. A targeted history to evaluate for thrombocytopenia, including explicit questions about the history of easy bruising, may have prompted earlier detection of thrombocytopenia prior to epidural placement in this case. Alternatively, requiring at least one platelet count level to have been recorded during the current pregnancy may reduce the risk of undetected thrombocytopenia and adverse events with epidural placement. A collaborative multidisciplinary approach including consultation with anesthesiology is needed to develop a hospital-wide protocol for the preanesthesia evaluation.

The trigger for the onset of ES in this patient was unclear. Cytokine activity in pregnancy may lead to increased autoimmunization against red blood cells [4], leading to higher rates of AIHA in pregnancy. About half of patients with ES have other immune disorders such as systemic lupus erythematosus, immunodeficiencies, or autoimmune lymphoproliferative disorders. These cases, referred to as secondary ES, may be more responsive to treatment [7] than primary ES, which is not associated with an underlying condition. At least one case report of a patient with SARS-CoV-2 infection and underlying immune dysregulation subsequently found to have ES has been described [8]. The onset of this patient's COVID-19 infection was unknown; however, preexisting COVID-19 infection leading to widespread immune activation prior to her initial admission may have served as a trigger for the new onset of ES. Her clinical course and robust treatment response are also concordant with secondary ES.

Alternatively, the use of immunosuppressive therapies including steroids and rituximab may have contributed to her development of COVID-19 pneumonia. Further studies are warranted to delineate the association of immunosuppressive agents used for ES with respiratory infections or whether infection serves as a potential trigger for ES in pregnancy.

Many autoimmune diseases, including warm AIHA, are associated with an increased risk of venous thromboembolic events. This risk is cited as 15%-33% [9] in warm AIHA. Additionally, thromboembolic events may be associated with IVIG, with added risk during pregnancy and the postpartum period. The use of prophylactic anticoagulation after delivery and systematic screening for venous thromboembolism in patients with ES is an area that may benefit from further studies.

Lessons to be incorporated from this case include the need to identify rare causes of anemia and thrombocytopenia in pregnancy such as ES and differentiate these from other causes more common in pregnancy. Intrapartum management of patients with ES highlights the need for the development of an institutional protocol for preanesthesia evaluation in the obstetric setting, perhaps with targeted history taking for signs of thrombocytopenia. In the postpartum period, systematic VTE screening and prophylaxis protocols for diseases associated with increased thrombotic risk may be beneficial.

## Figures and Tables

**Table 1:** Initial laboratory values.

Laboratory test	Value
Hemoglobin	7.1 g/dL^∗^
Hematocrit	22.1%^∗^
Mean corpuscular volume	74.7 fL^∗^
Mean corpuscular hemoglobin concentration	32.3 g/dL
Red blood cell distribution width	18.1%^∗^
Platelets	<10 K/*μ*L^∗^
White blood cell count	9.9 K/*μ*L
Prothrombin time	12.6 seconds
Partial thromboplastin time	27 seconds
International normalized ratio	0.98
Fibrinogen	388 mg/dL
Aspartate aminotransferase	16 IU/L
Alanine aminotransferase	7 IU/L
Total bilirubin	0.8 mg/dL
Direct bilirubin	0.1 mg/dL
Alkaline phosphatase	296 IU/L^∗^
Lactate dehydrogenase	263 IU/L^∗^
Ferritin	17 ng/mL^∗^
D-dimer	2.63 *μ*g/mL FEU^∗^
Iron	45 *μ*g/dL^∗^
Iron saturation	10%^∗^
Folate	12.4 ng/mL^∗^
Vitamin B12	136 pg/mL^∗^
Total iron-binding capacity	448 *μ*g/dL^∗^
Haptoglobin	<30.0 mg/dL^∗^
Reticulocyte percent	6.0%^∗^
Absolute reticulocyte count	149.4 K/*μ*L^∗^

^∗^Abnormal values.

## Data Availability

The data presented in this study pertained to only one patient, and relevant deidentified data is presented in the table included in the manuscript.

## References

[1] Jaime-Pérez J. C., Aguilar-Calderón P. E., Salazar-Cavazos L., Gómez-Almaguer D. (2018). Evans syndrome: clinical perspectives, biological insights and treatment modalities. *Journal of Blood Medicine*.

[2] Lefkou E., Nelson-Piercy C., Hunt B. J. (2010). Evans’ syndrome in pregnancy: a systematic literature review and two new cases. *European Journal of Obstetrics, Gynecology, and Reproductive Biology*.

[3] Parveen S., Mukhtar R., Shafee S., Mehmood R. (2019). Evans syndrome and pregnancy: a case report with literature review. *The Journal of the Pakistan Medical Association*.

[4] Suzuki H., Yamanoi K., Ogura J. (2019). A case of pregnancy complicated with Evans syndrome with sequential development of autoimmune warm antibody hemolytic anemia and idiopathic thrombocytopenic purpura. *Case Reports in Obstetrics and Gynecology*.

[5] Siddiqua S., Abbasi S., Fatema K. (2017). *Pregnancy with Evans syndrome – a rare case report*.

[6] American Society of Anesthesiologists Committee on Standards and Practice Parameters (2016). Practice guidelines for obstetric anesthesia: an updated report by the American Society of Anesthesiologists Task Force on Obstetric Anesthesia and the Society for Obstetric Anesthesia and Perinatology. *Anesthesiology*.

[7] Michel M., Chanet V., Dechartres A. (2009). The spectrum of Evans syndrome in adults: new insight into the disease based on the analysis of 68 cases. *Blood*.

[8] Wahlster L., Weichert‐Leahey N., Trissal M., Grace R. F., Sankaran V. G. (2020). COVID-19 presenting with autoimmune hemolytic anemia in the setting of underlying immune dysregulation. *Pediatric Blood & Cancer*.

[9] Audia S., Bach B., Samson M. (2018). Venous thromboembolic events during warm autoimmune hemolytic anemia. *PLoS One*.

